# Development of a machine learning model to predict low vision aid fitting for visually impaired patients

**DOI:** 10.3389/fmed.2025.1683484

**Published:** 2026-01-12

**Authors:** Bingfa Dai, Pengpeng Pei, Zunqi Kan, Hai Lan, Wenwen Ye, Yang Yu, Xuelan Chen, Yuyuan Yan, Ting Chen, Jianqing Zheng, Lijuan Huang, Jianmin Hu

**Affiliations:** 1 Department of Ophthalmology, The Second Affiliated Hospital of Fujian Medical University, Quanzhou, China; 2 Fujian Province University Engineering Research Center of Assistive Technology for Visual Impairment, Quanzhou, China; 3 Heji Hospital Affiliated of Changzhi Medical College, Changzhi, Shanxi, China; 4 Guang'anmen Hospital, China Academy of Chinese Medical Sciences, Beijing, China; 5 Quanzhou Institute of Equipment Manufacturing, Haixi Institute, Chinese Academy of Sciences, Jinjiang, Fujian, China; 6 Department of Radiation Oncology, The Second Affiliated Hospital of Fujian Medical University, Quanzhou, China

**Keywords:** AI-assistive device fitting, low-vision aids, machine learning, neural network model, random forest model

## Abstract

**Background:**

At present, the fitting of low-vision aids (LVA) for patients globally necessitates the intervention of highly skilled ophthalmologists and certified rehabilitation specialists. To mitigate this limitation, we employed machine learning algorithms to develop an artificial intelligence (AI)-based model for automated LVA fitting assistance.

**Patients and Methods:**

Clinical characteristics and diagnostic data from patients with low vision in southeastern China were collected between October 26, 2015, and October 6, 2021, to establish the training and test datasets. We developed and compared three machine learning models—Random Forest (RF), Deep Neural Network (DNN), and Logistic Regression (LR)—to predict prescriptions for three LVA categories selected based on compliance with the World Health Organization's basic specifications for assistive products: Distant Optical Visual aids (DOV), Near Electronic Visual aids (NEV), and Near Optical Visual aids (NOV). Hyperparameter optimization was conducted through four rounds of internal cross-validation. Following model training, the best-performing model was identified and subsequently validated on external data to assess its predictive accuracy and sensitivity.

**Results:**

The dataset comprised a total of 1,241 patients diagnosed with low vision. Our model displayed satisfactory performance in LVA fitting when evaluated on the test set. Comparative analysis revealed the RF model as the optimal choice, achieving area under the curve (AUC) values of 0.93 for DOV, 0.83 for NEV, and 0.91 for NOV. Furthermore, feature importance analysis derived from the RF model weights indicated that patient age, best-corrected visual acuity (BCVA of the left eye), and consultation year were the predominant factors influencing LVA fitting decisions across all three aid categories, while visual disability grade specifically impacted DOV prescriptions. In external validation involving 112 prospective cases, the model demonstrated performance comparable to that of a mid-career ophthalmologist (5 years' experience).

**Conclusion:**

This study identified significant associations between clinical characteristics and LVA prescription patterns. Leveraging historical LVA fitting data, we developed a machine learning-based decision support system capable of predicting optimal fittings for the three fundamental LVA categories. The proposed tool demonstrates potential for clinical application by generating data-driven prescription recommendations.

## Highlights

- The AI-assisted LVA fitting model achieved performance parity with ophthalmologists possessing over 15 years of clinical experience, validating its readiness for real-world clinical implementation.
- The study identified cataracts as the predominant cause of low vision in southeastern China, followed by fundus pathologies. Notably, a substantial proportion of patients required near optical visual aids (NOV), reflecting distinct regional visual rehabilitation needs.
- The AI model exhibited robust predictive performance for Distant Optical Visual aids (DOV), with Best Corrected Visual Acuity (BCVA) serving as the primary determinant—a finding that underscores the model's clinical utility in optimizing LVA prescriptions.

## 1 Introduction

Visual impairment presents a critical global health challenge, affecting approximately 1.3 billion people worldwide, including 300 million cases of clinically significant low vision (1). Notably, 90% of cases occur in less developed regions, highlighting an urgent need in those areas (1). China accounts for a substantial portion of this pandemic, with national surveys reporting 17 million visually impaired individuals—including 4.5 million blind and 12.5 million with moderate-to-severe vision loss (2, 3). Demographic projections suggest this situation will intensify with population aging trends, necessitating innovative intervention strategies (4). The 2022 Dynamic Survey of People with Disabilities quantifies this imperative, identifying nearly 4 million low-vision individuals requiring immediate assistive interventions (4).

The ramifications of visual impairment transcend mere sensory deficit, manifesting as multidimensional functional impairments (including dyslexia, impaired driving, mobility difficulties, and loss of independence) as well as substantial socioeconomic consequences (such as diminished workforce participation, elevated healthcare utilization, and educational attainment deficits) (5–9) can substantially ameliorate functional capacity and quality-adjusted life years (10), implementation challenges persist, particularly in developing nations (1). These challenges stem primarily from shortages of trained specialists and limited medical resources, resulting in grossly inadequate low-vision aids (LVA) fitting capacity and service coverage (11).

Previously, the Second Affiliated Hospital of Fujian Medical University has implemented an innovative community-based rehabilitation paradigm in southeastern China through a physician-led home visitation model, conducted in partnership with the China Disabled Persons' Federation (CDPF) (12). While this personalized approach demonstrated clinical efficacy, its generalizability remains constrained by prohibitive resource requirements, particularly for remote and rural populations.

The rapid advancement of artificial intelligence (AI) in healthcare presents new opportunities to address these challenges (12, 13) While ophthalmic diagnostics have embraced machine learning innovations, low-vision rehabilitation remains conspicuously underserved by computational approaches (14). Current LVA fitting methodologies rely heavily on traditional statistical approaches, with no internationally standardized clinical model available (15). Our preliminary investigations have established statistically significant correlations between AI-assisted LVA fitting and key outcome measures including visual function indices, rehabilitation demand profiles, and validated quality-of-life instrument scores (12).

This study aims to develop and validate a standardized, AI-powered model for LVA fitting to enable accurate, efficient rehabilitation service delivery. By employing neural network architectures, we aim to establish a clinically deployable predictive model capable of: (1) optimizing rehabilitation outcomes through precision fitting; (2) democratizing access to underserved populations; and (3) establishing the evidence-based international standard for LVA prescription. This paradigm-shifting approach holds particular promise for resource-constrained settings, potentially revolutionizing low-vision care delivery across rural China and analogous global health contexts. However, despite the rapid progress of AI in ophthalmic diagnostics, AI-based low-vision aid fitting has not yet been systematically developed or validated, highlighting a critical research gap that this study seeks to address.

## 2 Methods

**Figure 1:**
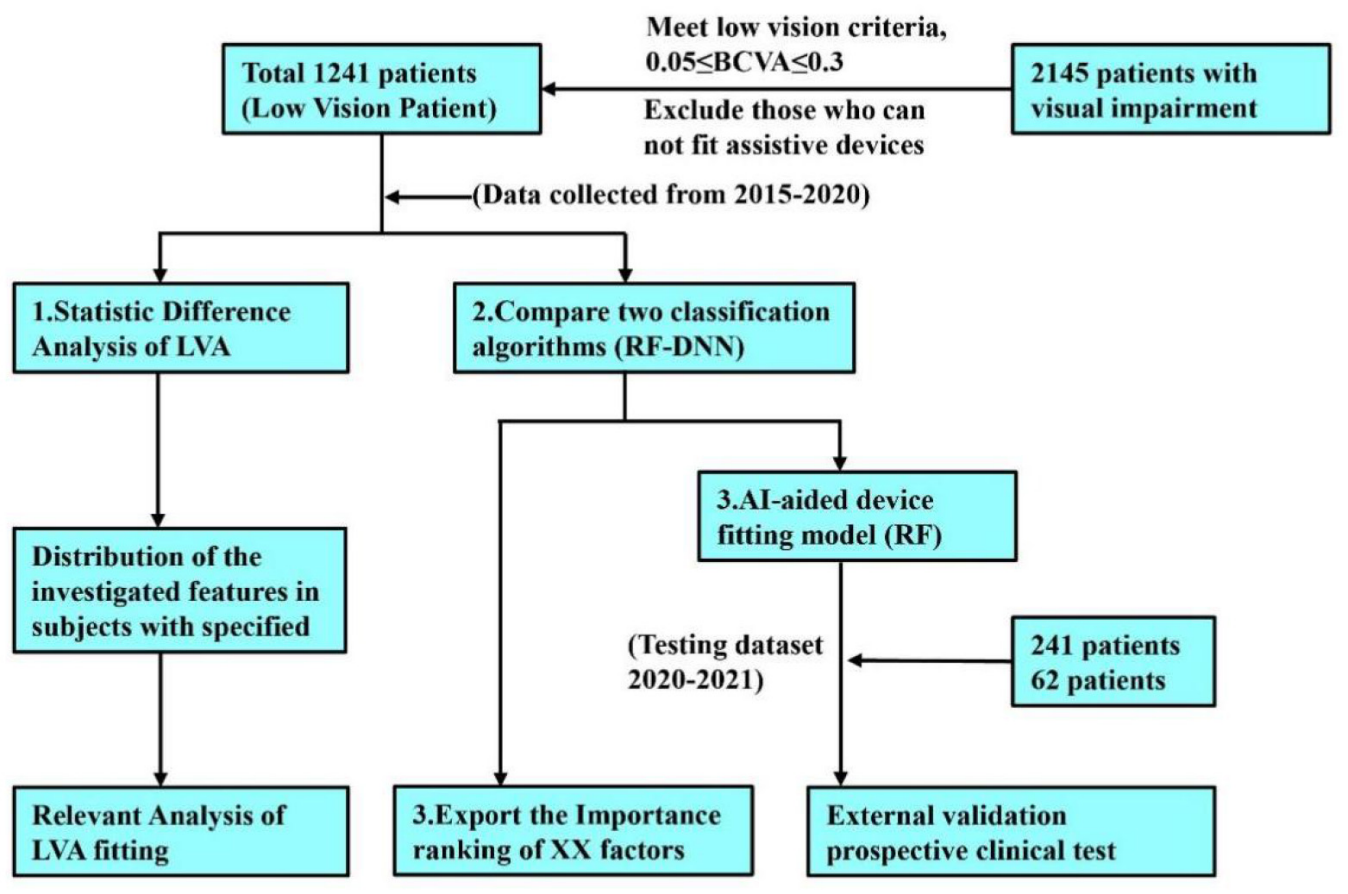
Study flowchart illustrating the outcall LVA fitting process and research methodology for patients with visual impairment.

The study protocol, including subject recruitment, predictive data collection, and machine learning model development, is presented in Figure 1.

### 2.1 Subject enrollment and ethics statement

From October 26, 2015, to October 6, 2021, the Visual Impairment Rehabilitation Instruction Center at the Second Affiliated Hospital of Fujian Medical University performed comprehensive visual assessments and LVA fittings for 2,645 visually impaired individuals across southeastern China (Fujian and Guangdong provinces). All assessments and LVA fittings were carried out at a single tertiary institution under a standardized clinical protocol, representing a single-center cohort that included multiple geographic regions. According to predefined inclusion and exclusion criteria, we enrolled 1,241 low-vision patients: (1) diagnosis conforming to WHO Low Vision and Blindness Criteria (16), and (2) capacity to complete LVA fitting. Exclusion criteria comprised: (1) cognitive or behavioral impairments affecting protocol compliance, and (2) comorbid severe disabilities (e.g., cerebral palsy or intellectual disability). This final cohort served as our dataset for predictive model development and validation.

The study protocol was approved by the Institutional Ethics Committee of the Second Affiliated Hospital of Fujian Medical University (Approval No. 170) and registered at ClinicalTrials.gov (NCT04919837). All data were anonymized and maintained with strict confidentiality throughout the study. Following detailed explanation of study procedures, written informed consent was obtained from all participants or their legal guardians (for minor patients), in full compliance with the Declaration of Helsinki principles. Each participant or their authorized representative provided signed consent documentation prior to enrollment.

**Figure 2:**
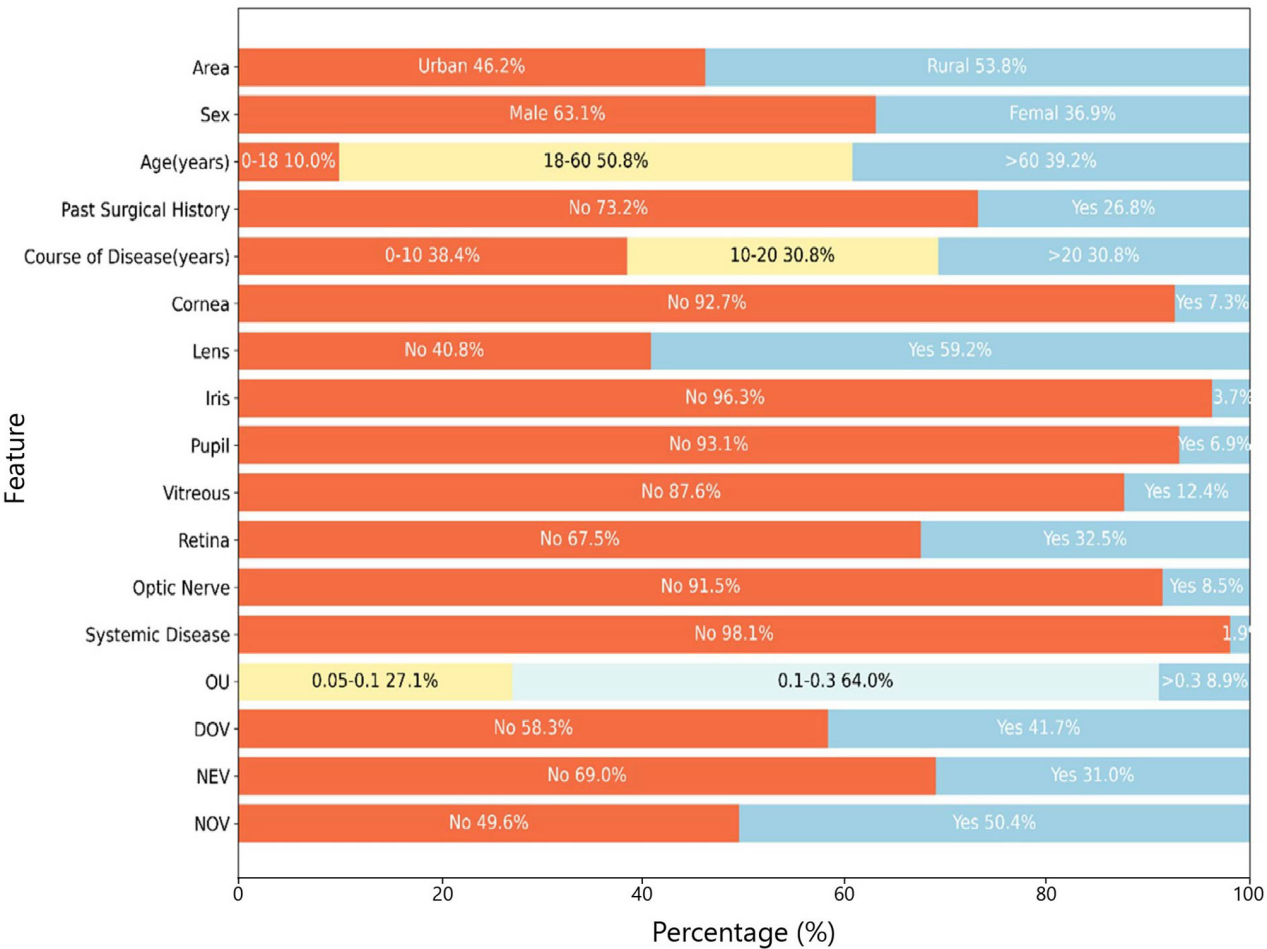
Demographic and clinical baseline characteristics of the study cohort. Distributions are shown for demographic factors, clinical history, ocular anatomy findings, binocular best-corrected visual acuity (BCVA-OU), and low-vision aid (LVA) prescription rates (DOV, NEV, NOV). Data represent percentages of the total cohort.

Demographic, pathological, diagnostic, and best-corrected visual acuity (BCVA) data were pooled for the 1,241 enrolled subjects (Table 1 and Figure 2). Candidate predictors (region, onset time, sex, age, course of the disease, cornea, lens, pupil, iris, vitreous, retina, optic nerve, brain and systemic etiology, disease diagnosis, visual impairment

**Table 1:** Baseline characteristics and prescription patterns of the study cohort (8:2 training-test split).

**Characteristic**	**Total cohort (*n* = 1,241)**	**Training set (*n* = 993, 80.0%)**	**Test set (*n* = 248, 20.0%)**	***P*-value**
**Demographics**				
Age, mean ± SD (years)	51.4 ± 20.8	51.8 ± 20.2	50.5 ± 22.1	0.456
Age range (years)	3–96	4–96	3–89	
**Sex**, ***n*** **(%)**				
Male	783 (63.1%)	625 (62.9%)	158 (63.7%)	0.912
Female	458 (36.9%)	368 (37.1%)	90 (36.3%)	
**Residence**, ***n*** **(%)**				
Urban	573 (46.2%)	459 (46.2%)	114 (45.9%)	0.938
Rural	668 (53.8%)	535 (53.9%)	133 (53.6%)	
**Past surgical history**, ***n*** **(%)**				
Yes	332 (26.8%)	266 (26.8%)	66 (26.6%)	0.724
No	909 (73.2%)	727 (73.2%)	182 (73.4%)	
**Disease duration**, ***n*** **(%)**				
0–10 years	477 (38.4%)	381 (38.4%)	96 (38.7%)	0.689
10–20 years	382 (30.8%)	302 (30.4%)	80 (32.3%)	
>20 years	382 (30.8%)	310 (31.2%)	72 (29.0%)	
**Ocular pathologies**, ***n*** **(%)**				
Cataract	580 (46.7%)	463 (46.6%)	117 (47.2%)	0.847
Retinopathy	838 (67.5%)	668 (67.3%)	170 (68.1%)	0.801
Lens lesions	736 (59.3%)	588 (59.2%)	149 (59.7%)	0.893
Glaucoma	156 (12.6%)	124 (12.5%)	32 (12.9%)	0.876
Age-related macular degeneration	186 (15.0%)	149 (15.0%)	37 (14.9%)	0.971
Diabetic retinopathy	210 (16.9%)	168 (16.9%)	42 (16.9%)	0.998
Corneal opacity	93 (7.5%)	74 (7.5%)	19 (7.7%)	0.925
**LVA prescription**, ***n*** **(%)**				
DOV	517 (41.7%)	413 (41.6%)	104 (42.0%)	0.902
NEV	385 (31.0%)	308 (31.0%)	77 (31.1%)	0.978
NOV	626 (50.4%)	501 (50.4%)	125 (50.4%)	0.999
**Supplementary interventions**, ***n*** **(%)**				
Lighting fixtures	371 (29.9%)	296 (29.8%)	75 (30.2%)	0.895
Absorption coatings	289 (23.4%)	230 (23.2%)	59 (23.8%)	0.831
Canes	114 (9.2%)	91 (9.2%)	23 (9.3%)	0.974

DOV, distant optical visual aids; NEV, near electronic visual aids; NOV, near optical visual aids.

### 2.2 Collection and labeling data on potential predictive factors

The study employed a rigorous data collection protocol in which two experienced ophthalmologists (each with over 15 years specializing in low vision rehabilitation) gathered comprehensive demographic and diagnostic information using standardized clinical tools including ETDRS visual acuity charts, Haag-Streit BX900 slit lamps, and Heine Beta 200 handheld funduscopic mydriasis devices. All collected data were systematically organized into four categories: (1) demographic characteristics covering geographic distribution (urban/rural), sex, age, and disease duration; (2) detailed ocular pathology documentation including corneal, pupillary, iris, lens, vitreous, retinal, optic nerve and systemic abnormalities; (3) diagnostic parameters incorporating BCVA measurements and specific diagnoses ranging from common conditions like cataracts and glaucoma to rare disorders such as Leber congenital amaurosis and retinitis pigmentosa; and (4) LVA prescriptions which were independently verified by two deputy senior ophthalmologists from provincial tertiary hospitals. Given the inherent subjectivity in LVA prescription decisions and the absence of definitive quantitative standards, we established an arbitration mechanism involving consultation with an International Organization for Rehabilitation of Low Vision expert to resolve any discrepancies between the evaluating ophthalmologists.

The final dataset comprised 1,241 carefully selected participants, with all collected variables—including geographic, temporal, demographic, ocular examination, systemic, diagnostic and visual function parameters—serving as candidate predictors for our machine learning analysis. The outcome variables consisted of professionally prescribed LVA categorizations—DOV (including 4 × /6 × optical and TV magnifiers), NEV (comprising 3.4″/4.3″ electronic devices), and NOV (incorporating various magnifier types from paperweight to eyeglass-mounted)—that were binary-coded according to WHO specifications while accommodating multiple concurrent prescriptions per patient. This comprehensive, clinically validated dataset formed the foundation for our predictive modeling approach, with all LVA classifications reflecting real-world rehabilitation practice standards.

### 2.3 Development of machine learning models

The predictive modeling framework incorporated clinically relevant input features, including key demographic characteristics (age, gender, region), functional visual parameters BCVA for the right eye (OD), left eye (OS), and both eyes (OU); visual disability grade), disease-specific factors (diagnosis, disease duration), and anatomical assessments. Prior to model training, missing values for continuous variables (e.g., BCVA, age) were imputed using median values, while missing categorical variables (e.g., diagnosis, sex) were imputed with the most frequent category. Categorical features were transformed using one-hot encoding, and continuous features were standardized to have zero mean and unit variance (17). From the total cohort of 1,241 participants, we implemented a randomized 80:20 split, allocating 1,000 cases to the training set and 241 to the independent test set. The modeling architecture employed three machine learning approaches to systematically investigate the relationship between clinical features and LVA prescription patterns: Logistic Regression (LR) was included as a simple, interpretable baseline model, while Random Forest (RF) and Deep Neural Network (DNN) algorithms were selected for their complementary strengths. RF was chosen for its high performance with structured clinical data, robustness to outliers and non-linear relationships, and, critically, its inherent ability to provide feature importance measures for clinical interpretability (18). DNN was investigated to assess its capacity to capture complex, high-level feature interactions that might be missed by other models (19). Separate binary classification models developed for each LVA category (DOV, NEV, NOV).

Model development incorporated a rigorous 4-fold cross-validation protocol on the training data to optimize hyperparameters and evaluate algorithm performance. For the RF implementation, we tuned the number of decision trees, while the DNN architecture required optimization of hidden layer configuration (number and size of layers) and regularization parameters (alpha values) to prevent overfitting. To address class imbalance in the training data, we applied random oversampling of minority classes until achieving parity between positive and negative samples, thereby ensuring balanced learning and preventing model bias toward majority classifications. This resampling strategy was implemented separately for each LVA category during the cross-validation phase to maintain the integrity of performance evaluation while optimizing predictive accuracy across all prescription types. To further enhance model interpretability, Random Forest feature importance was quantified using the Mean Decrease in Gini impurity (20), and permutation importance was additionally computed to assess the robustness of feature rankings.

### 2.4 Clinical validation: physician vs. AI model performance

To rigorously assess the clinical applicability of our prediction model, we conducted an external validation study involving 112 prospectively enrolled low vision patients randomly selected from distinct clinical populations across Guangdong and Fujian provinces. These patients were recruited from hospitals independent of the training cohort and from separate time periods, with no overlap of subjects, ensuring the external validation was fully independent. Under the supervision of the Visual Rehabilitation Guidance Center at the Second Affiliated Hospital of Fujian Medical University, we compared the model outputs with prescriptions made by ophthalmologists to evaluate both prescription accuracy (defined as agreement with final expert-prescribed LVAs) and patient-reported satisfaction metrics. The comparing ophthalmologists (with 5 and 15 years of experience respectively) were blinded to the model outputs during their prescription decisions to prevent assessment bias. To ensure robust statistical analysis, we implemented a repeated sampling approach with four iterations of bootstrap resampling (with replacement), which effectively minimized potential sampling variability while providing stable performance estimates.

### 2.5 Statistical analysis

The machine-learning models were implemented using Scikit-learn version 0.24.2, and the evaluation was performed using Python 3.7. We drew a chord diagram to visualize the connection between diagnosis and LVA prescription. The receiver operating characteristic (ROC) curve was drawn by plotting sensitivity vs. 1-specificity at all classification thresholds, which represented the model capacity in predicting LVA. The following methods were used to evaluate model performance: area under the receiver operating curve (AUC), accuracy, sensitivity $\left(\frac{TP}{TP+FN}\right)$, specificity $\left(\frac{TN}{TN+FP}\right)$ +, and FPF1 score, which is the harmonic mean of sensitivity and precision$\left(\frac{TP}{TP+FP}\right)$. The 95% confidence intervals (CIs) were the Wald CIs for AUC, accuracy, sensitivity, specificity, and F1 score, which were calculated using bootstrapping with 1,000 replicates. Statistical analysis and chord diagrams were generated using R software (version 4.1.0).

## 3 Results

### 3.1 Cohort characteristics and prescription patterns

**Figure 3:**
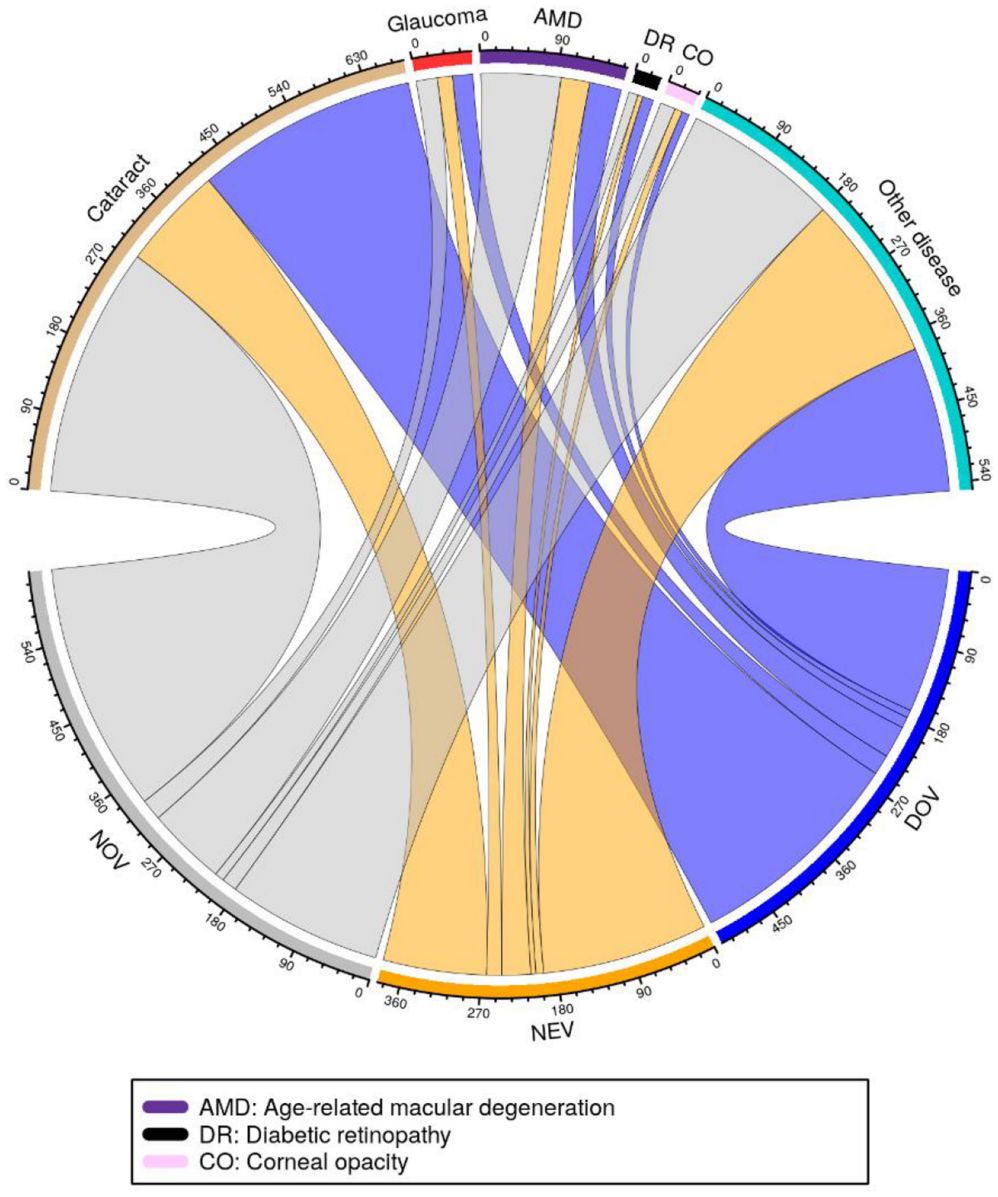
Correspondence between clinical diagnoses and prescribed low-vision devices among study participants. The chord diagram visualizes the associations between primary clinical diagnoses (including glaucoma, age-related macular degeneration-AMD, diabetic retinopathy-DR, corneal opacity-CO, and other conditions) and corresponding prescriptions of DOV, NEV, and NOV visual aids. Line connections and widths represent the strength of relationship between specific diagnoses and aid categories.

The analysis included 1,241 patients randomly divided into training (*n* = 1,000, 80.6%) and test (*n* = 241, 19.4%) sets, with comparable age distributions (training set: 52 ± 20 years, range 4–96; test set: 49 ± 23 years, range 3–89). The cohort displayed a slight rural predominance (53.8%, 668/1,241) vs. urban participants (46.2%, 573/1,241). Ocular pathologies were predominantly cataracts (46.7%, 580/1,241), retinopathy (67.5%, 838/1,241), and lens lesions (59.3%, 736/1,241), with comprehensive statistical visualization provided in Figure 3.

Prescription analysis revealed all patients received LVA, with NOV being most frequent (50.4%, 626/1,241), followed by DOV (41.7%, 517/1,241) and NEV (31.0%, 385/1,241). Lighting fixtures (29.9%), absorption coatings (23.4%), and canes (9.2%) comprised the remaining interventions. These categories are not mutually exclusive, as some patients were prescribed multiple types of aids. Diagnostic-specific patterns emerged clearly: cataract patients primarily required NOV (30.2%) and DOV (27.6%), while glaucoma cases showed preference for lighting fixtures (39.1%). Age-related macular degeneration correlated strongly with NOV prescriptions (40.0%), contrasting with diabetic retinopathy's association with ROV (34.2%). Corneal opacity patients demonstrated dual peaks for NOV (29.5%) and lighting fixtures (24.6%), while other conditions maintained relatively even aid distributions. These findings demonstrate the critical relationship between underlying pathology and visual rehabilitation requirements, with distinct prescription profiles for each major diagnostic category.

### 3.2 Performance evaluation of LVA fitting models

**Figure 4:**
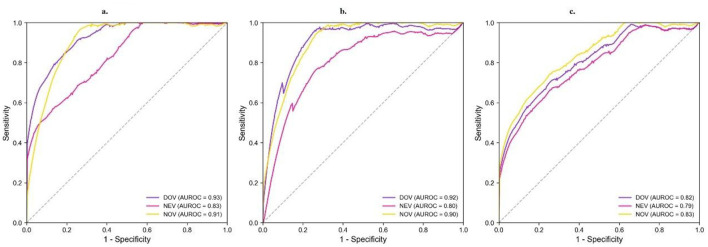
ROC curves for predicting LVA prescriptions. Performance of **(a)** RF, **(b)** DNN, and **(c)** LR models in classifying three LVA types: DOV (blue), NEV (purple), and NOV (yellow). AUROC values are shown for each curve. The gray dashed line indicates random guessing (AUROC = 0.5).

To address class imbalance in the training data, we implemented random oversampling prior to model development. Through systematic four-fold cross-validation, we optimized hyperparameters and evaluated three machine learning approaches—LR as a baseline, RF, and DNN - across all three LVA categories. As detailed in Table 2 and Figure 4 comparative analysis revealed RF models demonstrated superior overall performance, with AUC values of 0.93 (DOV), 0.83 (NEV), and 0.91 (NOV) compared to DNN's 0.92 (DOV), 0.80 (NEV), and 0.90 (NOV). The baseline LR model consistently demonstrated substantially lower performance across all metrics and LVA categories, with AUC values of 0.82 (DOV), 0.79 (NEV), and 0.83 (NOV), confirming that the non-linear relationships captured by the RF and DNN models were critical for this prediction task. However, the F1 score analysis presented an interesting divergence: while RF maintained strong specificity and balanced classification across both positive and negative samples, DNN models achieved higher sensitivity, particularly for positive cases. This performance characteristic likely stemmed from our F1 score optimization during cross-validation, which enabled the more complex DNN architecture to excel in recall-oriented prediction tasks. Notably, both algorithms showed clinically acceptable performance across all LVA categories, with RF exhibiting particular strength in comprehensive classification (as reflected in AUC) and DNN demonstrating advantages in positive case identification (as indicated by F1 scores). These complementary performance profiles suggest potential value in ensemble approaches that could leverage the strengths of both methodologies.

**Table 2:** Performance of models on test set.

**Category**	**Model**	**Accuracy (95% CI)**	**Sensitivity (95% CI)**	**Specificity (95% CI)**	***F*1 Score (95% CI)**	**AUROC (95% CI)**
DOV	RF	0.78 (0.73–0.83)	0.61 (0.52–0.69)	0.98 (0.96–1.01)	0.75 (0.68–0.82)	0.93 (0.90–0.96)
	DNN	0.88 (0.84–0.93)	0.97 (0.94–1.00)	0.78 (0.70–0.86)	0.90 (0.86–0.94)	0.92 (0.89–0.95)
	LR	0.59 (0.53–0.65)	0.52 (0.43–0.61)	0.66 (0.58–0.74)	0.56 (0.48–0.64)	0.82 (0.77–0.87)
NEV	RF	0.79 (0.74–0.84)	0.50 (0.40–0.59)	0.99 (0.98–1.01)	0.66 (0.57–0.75)	0.83 (0.78–0.88)
	DNN	0.73 (0.67–0.78)	0.95 (0.90–0.99)	0.58 (0.50–0.66)	0.74 (0.67–0.80)	0.80 (0.75–0.85)
	LR	0.65 (0.59–0.71)	0.60 (0.50–0.70)	0.70 (0.62–0.78)	0.62 (0.53–0.71)	0.79 (0.74–0.84)
NOV	RF	0.87 (0.83–0.91)	0.83 (0.76–0.90)	0.90 (0.85–0.95)	0.84 (0.79–0.90)	0.91 (0.88–0.94)
	DNN	0.87 (0.83–0.92)	0.76 (0.68–0.84)	0.96 (0.92–0.99)	0.83 (0.78–0.89)	0.90 (0.87–0.93)
	LR	0.60 (0.54–0.66)	0.58 (0.49–0.67)	0.62 (0.53–0.71)	0.59 (0.52–0.66)	0.83 (0.78–0.88)

### 3.3 Feature importance analysis

**Figure 5:**
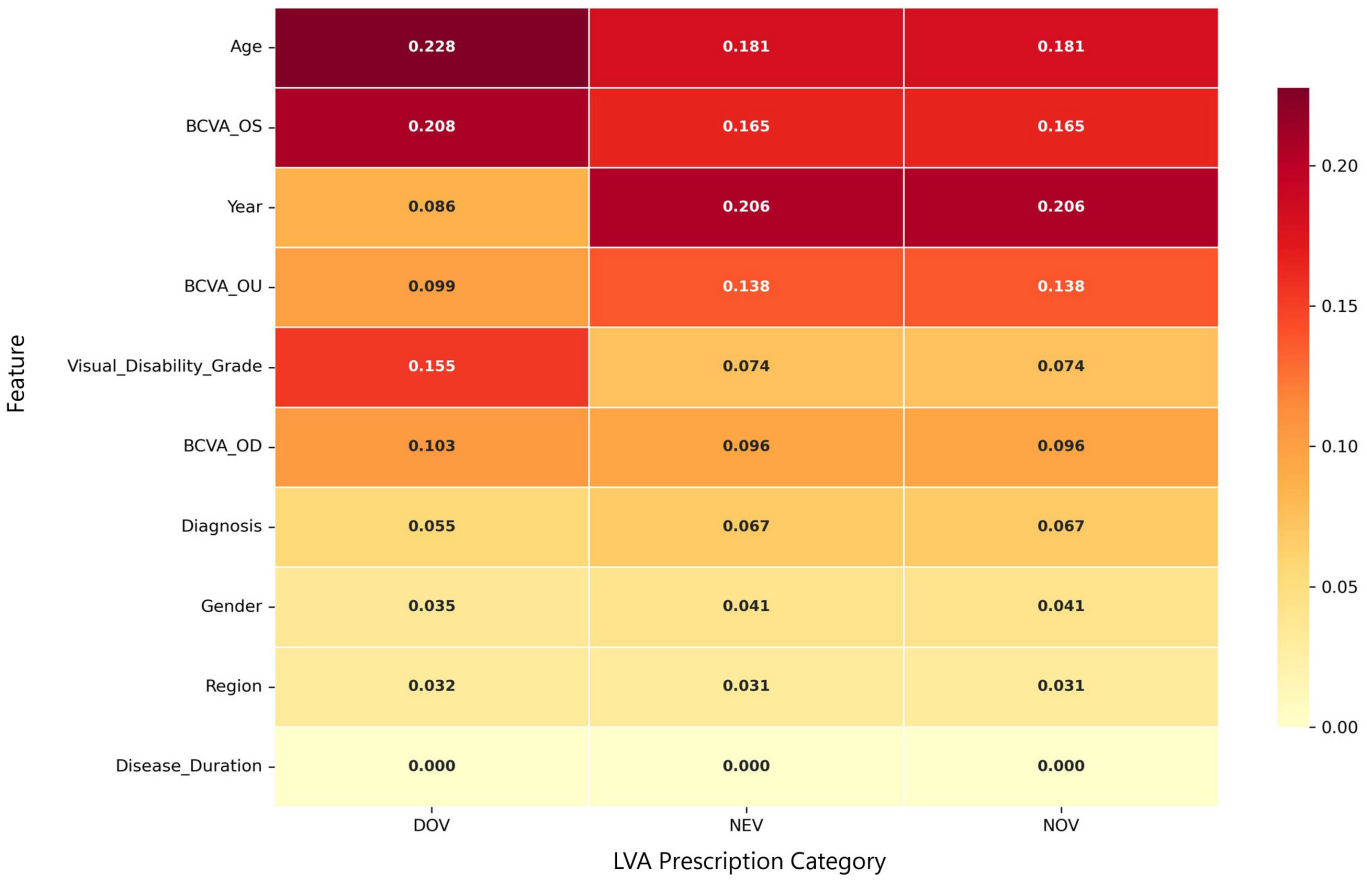
Heatmap of feature importance from the random forest models. The plot displays the importance weights of key clinical and demographic features for predicting prescriptions of the three LVA categories (DOV, NEV, NOV). The color intensity of each cell corresponds to the feature's importance score, with the gradient bar (right) indicating the value range from low (pale yellow) to high (dark red).

To improve model interpretability, we analyzed feature importance weights in the RF model. As detailed in Table 3 and Figure 5, the analysis revealed that patient age and BCVA of the OS were consistently among the top three most influential features across all three LVA categories. Notably, the specific ranking of top features varied by aid type: for DOV, age was the primary predictor, followed by BCVA_OS and visual disability grade; for both NEV and NOV, the year of consultation was the most important feature, followed by age and BCVA_OS. Furthermore, visual impairment grade was a key determinant for DOV prescriptions, while disease duration was identified as the least influential factor across all categories.

**Table 3:** Feature importance rankings derived from the random forest models for predicting LVA prescriptions.

**Rank**	**DOV**		**NEV**		**NOV**	
	**Feature**	**Importance**	**Feature**	**Importance**	**Feature**	**Importance**
1	Age	0.228	Year	0.206	Year	0.206
2	BCVA_OS	0.208	Age	0.181	Age	0.181
3	Visual disability grade	0.155	BCVA_OS	0.165	BCVA_OS	0.165
4	BCVA_OD	0.103	BCVA_OU	0.138	BCVA_OU	0.138
5	BCVA_OU	0.099	BCVA_OD	0.096	BCVA_OD	0.096
6	Year	0.086	Visual disability grade	0.074	Visual disability grade	0.074
7	Diagnosis	0.055	Diagnosis	0.067	Diagnosis	0.067
8	Gender	0.035	Gender	0.041	Gender	0.041
9	Region	0.032	Region	0.031	Region	0.031
10	Disease duration	0.000	Disease duration	0.000	Disease duration	0.000

DOV, distant optical visual aids; NEV, near electronic visual aids; NOV, near optical visual aids; BCVA, best-corrected visual acuity; OS, left eye; OD, right eye; OU, both eyes.

### 3.4 External clinical validation

For external validation, we implemented a rigorous comparative assessment between the AI model and clinical experts. Patient data were collected prospectively through an online platform, with an expert panel establishing the gold standard prescription for each case. These reference standards enabled direct comparison between the AI model's recommendations and those of qualified ophthalmologists (with 15- and 5-years' experience respectively) at a tertiary visual rehabilitation center.

**Figure 6:**
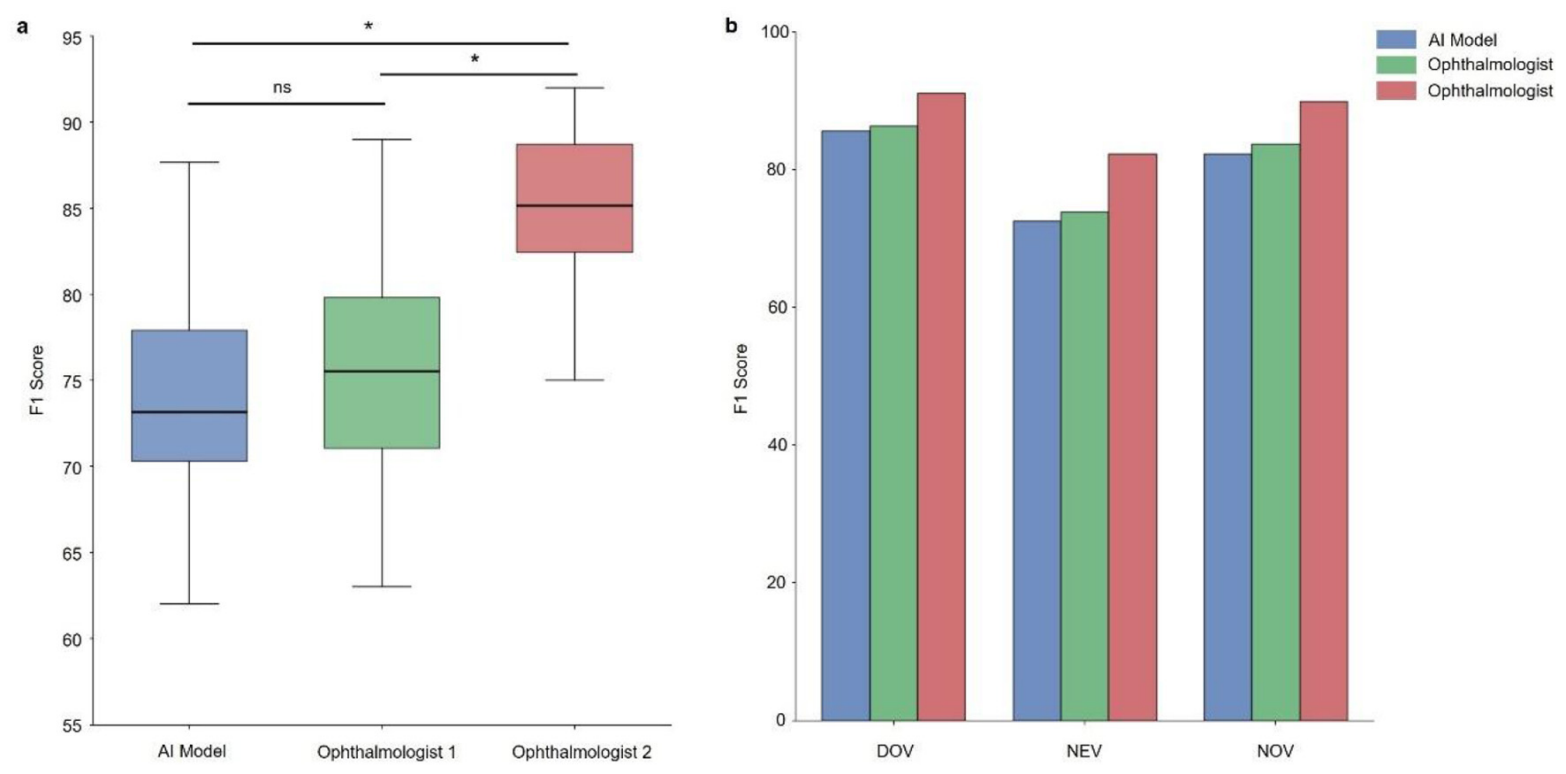
Comparative performance evaluation between the AI model and ophthalmologists in low-vision aid (LVA) fitting. **(a)** The F1 scores of the AI model and two ophthalmologists (with 5 and 15 years of low-vision rehabilitation experience, respectively) for LVA fitting across 30 complex external validation cases. **(b)** Performance breakdown by LVA category, showing the F1 scores of the AI model, Ophthalmologist 1 (5 years' experience), and Ophthalmologist 2 (15 years' experience) for predicting DOV, NEV, and NOV. The symbol * indicates a statistically significant difference between groups.

The evaluation incorporated a challenging test set of 30 complex low-vision cases representing clinical scenarios not encountered during model training. Statistical analysis revealed the AI model's performance showed significant divergence from the senior ophthalmologist (15 years' experience; *p* < 0.05) but comparable results to the mid-career specialist (5 years' experience; *p* > 0.05). Across three independent trials, the model demonstrated consistent F1 scores (range: 62.12–91.04) that closely paralleled physician performance (range: 63.45–92.08) for all three LVA categories. These results, detailed in Figure 6, confirm the model's clinical utility in assistive device fitting, approaching the performance of mid-level specialists in most prescription scenarios while highlighting areas for further refinement to match top-tier clinical expertise.

## 4 Discussion

In this study, we revealed the association between 10 clinical features and three categories of LVA prescription and verified the capability of the AI-aided model in generating LVA fitting suggestions. The feature importance analysis further illuminated these associations, quantitatively demonstrating that core clinical measures like age and BCVA were the primary drivers of the model's predictions, which aligns well with established rehabilitation principles (21). The model's performance on the clinical test set demonstrated that the AI-aided LVA fitting model could achieve results approaching those of mid-level specialists. This performance level suggests the model's potential as a valuable decision-support tool in primary care settings or regions with limited access (22) to highly specialized ophthalmological expertise, potentially improving the efficiency and accessibility of low-vision rehabilitation services. However, external validation was limited to 30 complex cases, and the generalizability of the model across different populations has not yet been fully established, indicating that practical, widely generalizable clinical application remains to be confirmed. We explored a community-based rehabilitation system for people with low vision in Fujian and Guangdong provinces, China, which is similar to many rural areas in the country, using a low-vision aid fitting program. This model has the advantages of convenience and easy generalization and is expected to address the shortage of professionals in remote and underdeveloped areas.

Currently, the clinical application prospects of machine learning or statistical models in LVA fitting are unclear worldwide (15). It has been reported that patients can purchase LVA online, and visually impaired people can use smartphones, pads, or e-book readers to meet their reading needs. However, past studies lacked experience in big data analysis, patient satisfaction was low, and the rate of LVA abandonment was high after the fitting program was implemented (23). Novel digital health solutions could help overcome these clinical barriers by supporting timely diagnosis and referral (24). This model can be used for rapid LVA screening in China to facilitate the development of products targeted by LVA manufacturers. It was integrated into the cloud server of the Assistive Device Adaptation Center of the China Disabled Persons' Federation for online LVA fitting for patients who could not fit the LVA onsite. In the future, our model may be embedded into an LVA vending machine, thereby achieving integration of the LVA fitting process.

Our model exhibited satisfactory performance in predicting DOV, indicating the feasibility of using an AI-aided model for LVA fitting. DOV fitting was mainly determined by BCVA. This is strongly corroborated by our feature importance analysis, where BCVA_OS was the second most critical feature for DOV prediction. The comparatively lower performance for NEV and NOV may be attributable to several factors: the smaller number of positive cases in the training data (data imbalance) (25), the greater complexity and variability of the devices, and differences in patients' visual functions and daily usage requirements. Intriguingly, the feature ranking revealed that 'Year' was the top predictor for both NEV and NOV, suggesting that temporal factors (26) like evolving technology or clinical protocols may influence these prescriptions—a complex relationship that purely clinical models might not capture effectively. These factors could have contributed to reduced predictive accuracy for NEV and NOV compared to DOV. Additionally, the higher internal AUC values compared to external validation results suggest potential overfitting, which may have limited the model's generalizability. Future work with larger and more diverse datasets, as well as additional regularization or ensemble strategies (21), may help mitigate overfitting and improve external performance. Our study found that cataracts were the main cause of low vision in southeast China, which is consistent with other reports, followed by fundus lesions (27). We also found that patients with low vision had the highest proportion of NOV, followed by DOV and then NEV. This was because people with low vision came from both economically developed cities and economically underdeveloped villages and towns. China is a vast country with a significant gap between the rich and poor, and most people with low vision are from rural areas. The rehabilitation demand of these patients is still primarily for near vision; however, the proportion of patients requiring distance vision is also increasing significantly (28). Currently, patients with low vision in China mainly learn about news and current affairs by watching TV; hence, optical aids play an important role. This may explain the popularity of optical aids. Contrary to previous studies, the proportion of NEV used in the present study was high. Compared to NOV, NEV has a higher magnification and the advantage of adjusting various contrast sensitivities. With the improvement in economic levels and free LVA fitting services, NEV is becoming increasingly popular, so their fitting proportion is also high (29). With the increasing proportion of patients with fundus diseases and low vision, many of whom have visual field impairments, the coordination rate of electronic visual aids is also increasing (30).

Our model demonstrated good prediction accuracy for most basic types of LVA; however, there is still a gap compared to professional ophthalmologists. There are two possible reasons for this. One is the data sample itself; these two types of LVA had a low proportion of positive people and could not reflect the true distribution of the data sample, resulting in model overfitting. Additionally, these two types of LVA were related to the daily needs of patients from a usage perspective. In future research, we plan to address these issues by 1. Collecting more data and increasing the number of training samples; 2. Optimizing the algorithm model according to data imbalance, such as increasing the weight loss of the category with fewer samples.

### 4.1 Limitations

While this multicenter study provides valuable insights into AI-assisted LVA fitting, several limitations should be acknowledged. First, despite drawing from multiple centers, our sample size (*n* = 1,241) remains modest compared to other medical AI studies, potentially limiting generalizability. Second, the exclusively Chinese cohort (single ethnicity) may not fully represent global low vision populations, suggesting caution when applying these findings to other demographic groups. Third, our retrospective design could not account for post-fitting outcomes such as device abandonment rates. Future studies should incorporate longitudinal follow-up of non-adherent patients to identify modifiable factors affecting LVA acceptance. Finally, while our model demonstrated good performance, prospective comparative trials in real-world clinical settings are needed to fully evaluate its practical utility against human specialists across diverse practice environments.

## 5 Conclusions

This study successfully established clinically meaningful associations between key ophthalmic parameters and LVA prescription patterns, enabling development of a machine learning-based decision support tool for three fundamental LVA categories. Our AI model achieved performance approaching that of experienced clinicians, demonstrating particular strength in DOV prediction while identifying BCVA as the most influential factor. These findings support the feasibility of AI-assisted LVA fitting systems; however, the model is not yet equivalent to clinical judgment or ready for deployment. Future enhancements could increase versatility by incorporating patient complaints and feedback, as the current model primarily replicates ophthalmologists' or institutional dispensing patterns. Further optimization through expanded multicenter collaborations, prospective validation, increased sample diversity, real-world usability testing, and adaptive learning mechanisms will be crucial for translating this technology into routine clinical practice and improving access to quality low vision rehabilitation services globally.

## Data Availability

The original contributions presented in the study are included in the article/supplementary material, further inquiries can be directed to the corresponding author/s.
